# PReS-FINAL-2292: Hypogonadism and osteoporosis among adolescents with systemic lupus erythematosus

**DOI:** 10.1186/1546-0096-11-S2-P282

**Published:** 2013-12-05

**Authors:** D El-Ghoneimy, K Awwaad, E El-Hadidy, A Abdel-Latif

**Affiliations:** 1 Pediatric Allergy, Immunology and Rheumatology, Cairo, Egypt; 2 Clinical Pathology, Ain Shams University, Cairo, Egypt

## Introduction

Children and adolescents with systemic lupus erythematosis (SLE) are at risk for osteopenia and osteoporosis. Sex hormones protect against bone loss and as SLE itself and the immunosuppressive drugs given might interfere with normal puberty, this could add to the risk of bone loss among these patients.

## Objectives

To evaluate the frequency of osteoporosis and hypogonadism among adolescents with SLE and how far the two conditions co-exist. The effect of disease characteristics and immunosuppressive drugs on both conditions are studied as well.

## Methods

Thirty-six adolescents with SLE were evaluated to determine the characteristics of the disease. SLE disease activity index (SLEDAI) was used to assess SLE status. Beside routine laboratory investigations of SLE, measurement of follicle stimulating hormone(FSH), leutinizing hormone (LH) and estrogen (E2) for females or testosterone for males before and 4 hours after LH releasing hormone analogue (0.1 ml) injection. Dual emission X-ray absorptiometery (DEXA) scan was done for all patients.

## Results

Eighteen (50%) patients had low bone mass density(BMD), of these patients, 11 (31%) had osteoporosis (BMD < -2 Z-score) and 7(19%) had osteopenia (BMD <-1 and >-2 Z-score). Fifteen (42%) patients had hypogonadism: 12 (33%) had secondary hypogonadism and 3 (9%) had primary hypogonadism. The frequency of hypogonadism was comparable among patients with osteopenia/osteoporosis and those with normal BMD (p > 0.05). The age of the studied patients with osteopenia/osteoporosis had significant negative correlation with Z-score of DEXA scan (p < 0.05). Longer duration of SLE was associated with higher frequency of osteopenia/osteoporosis as well as hypogonadism among the studied patients (p = 0.01). While patients with osteopenia/osteoporosis had significantly higher SLEDAI as compared to those with normal BMD (p = 0.01), SLEDAI was comparable among patients with hypogonadism and those with normal gonadal function (p > 0.05). A significant negative correlation was found between the cumulative dose of steroids and Z-score of DEXA scan among patients with osteopenia and osteoporosis (p = 0.01). Patients with hypogonadism had received comparable cumulative doses of steroids and cyclophosphamide to those with normal gonadal function (p > 0.05).

## Conclusion

Osteopenia/osteoporosis and hypogonadism are common co-morbid conditions among adolescents with SLE. Lupus flare and steroids seem to affect BMD rather than gonadal function. Whether hypogonadism adversely affect BMD, this remains to be verified on a larger scale.

## Disclosure of interest

None declared

